# Kidney outcomes using a sustained ≥40% decline in eGFR: A meta‐analysis of SGLT2 inhibitor trials

**DOI:** 10.1002/clc.23665

**Published:** 2021-06-15

**Authors:** David Z. I. Cherney, Samuel Dagogo‐Jack, Darren K. McGuire, Francesco Cosentino, Richard Pratley, Weichung J. Shih, Robert Frederich, Mario Maldonado, Jie Liu, Shuai Wang, Christopher P. Cannon, the VERTIS CV Investigators

**Affiliations:** ^1^ Division of Nephrology University of Toronto Toronto Ontario Canada; ^2^ Division of Endocrinology, Diabetes and Metabolism University of Tennessee Health Science Center Memphis Tennessee USA; ^3^ Division of Cardiology University of Texas Southwestern Medical Center, and Parkland Health and Hospital System Dallas Texas USA; ^4^ Unit of Cardiology Karolinska Institute and Karolinska University Hospital Stockholm Sweden; ^5^ AdventHealth Translational Research Institute Orlando Florida USA; ^6^ Department of Biostatistics and Epidemiology Rutgers School of Public Health and Rutgers Cancer Institute of New Jersey New Brunswick New Jersey USA; ^7^ Clinical Development and Operations Research and Development, Pfizer Inc. Collegeville Pennsylvania USA; ^8^ Diabetes and Endocrinology MSD Limited London UK; ^9^ Global Product Development Statistics Merck & Co., Inc. Kenilworth New Jersey USA; ^10^ Pfizer Inc. Groton Connecticut USA; ^11^ Cardiovascular Division, Brigham and Women's Hospital Harvard Medical School Boston Massachusetts USA

**Keywords:** kidney disease, kidney failure, meta‐analysis, randomized clinical trials, SGLT2 inhibitor, type 2 diabetes mellitus

## Abstract

**Background:**

A recent meta‐analysis of sodium–glucose cotransporter 2 (SGLT2) inhibitor outcome trials reported that SGLT2 inhibitors were associated with reduction in the risk of adverse composite kidney outcomes, with moderate heterogeneity across the trials; however, the endpoints were defined differently across the trials.

**Hypothesis:**

The apparent heterogeneity of the meta‐analysis of kidney composite outcomes of SGLT2 inhibitor trials will be substantially reduced by using a consistent assessment of sustained ≥40% decline in eGFR/chronic kidney dialysis/transplantation/renal death across trials.

**Methods:**

We performed a meta‐analysis of kidney composite outcomes from the four SGLT2 cardiovascular outcome trial programs conducted in general type 2 diabetes mellitus populations, which included, as a surrogate of progression to kidney failure, a sustained ≥40% decline in eGFR along with kidney replacement therapy and kidney death. The trials assessed were VERTIS CV (NCT01986881), CANVAS Program (NCT01032629 and NCT01989754), DECLARE‐TIMI 58 (NCT01730534), and EMPA‐REG OUTCOME (NCT01131676).

**Results:**

Data from the trials comprised 42 516 individual participants; overall, 998 composite kidney events occurred. SGLT2 inhibition was associated with a significant reduction in the kidney composite endpoint (HR 0.58 [95% CI 0.51–0.65]) and with a highly consistent effect across the trials (Q statistic p = .64; *I* ^2^ = 0.0%).

**Conclusions:**

Our meta‐analysis highlights the value of using similarly defined endpoints across trials and supports the finding of consistent protection against kidney disease progression with SGLT2 inhibitors as a class in patients with type 2 diabetes mellitus who either have established atherosclerotic cardiovascular disease or are at high cardiovascular risk with multiple cardiovascular risk factors.

## 1 INTRODUCTION

Sodium–glucose cotransporter 2 (SGLT2) inhibitors are medications initially developed to manage hyperglycemia in type 2 diabetes. Results from cardiovascular outcome trials (CVOTs) demonstrated that some of these agents significantly reduce the risk of major adverse cardiovascular and kidney events.^1^ In these trials, composite kidney outcomes were reported, comprising hard kidney outcomes of kidney death and kidney failure (kidney replacement therapy and end‐stage kidney disease), as well as surrogate measures of progression to kidney failure; i.e., doubling of serum creatinine (corresponding to a 57% decline in estimated glomerular filtration rate [eGFR]), or a 40% to 50% sustained decline in eGFR from baseline. One of the secondary endpoints from the ertugliflozin cardiovascular outcome trial (VERTIS CV) was a composite kidney outcome comprising doubling of serum creatinine/kidney dialysis/transplantation/kidney death, which had a hazard ratio (HR) (95% confidence interval [CI]) of 0.81 (0.63–1.04).^2^ A recent meta‐analysis of SGLT2 inhibitor outcome trials reported that SGLT2 inhibitors were associated with reduction in the risk of adverse composite kidney outcomes (HR 0.62, 95% CI 0.56–0.70), with moderate heterogeneity across the trials (Q statistic p = .09; *I* ^2^ = 49.7%).^1^

Previous meta‐analyses of kidney outcomes from SGLT2 inhibitor trials have not, however, used uniform definitions of “significant kidney function decline”, which have differed between the trials.^1^ The importance of using consistent definitions of eGFR decline has been emphasized by recent expert consensus statements, as thresholds for kidney function loss must be clinically relevant and also feasible over the 2–4 year time frame of clinical outcome trials.^3^, ^4^ Accordingly, our aim was to examine kidney composite outcome data from four SGLT2 inhibitor CVOTs using a uniform sustained ≥40% eGFR decline definition.^3^, ^4^

## 2 METHODS

The VERTIS CV trial (NCT01986881; protocol MK‐8835‐004) assessed the effects of ertugliflozin versus placebo in patients with type 2 diabetes and established atherosclerotic cardiovascular disease (ASCVD). The study was conducted in accordance with principles of Good Clinical Practice and was approved by the appropriate institutional review boards and regulatory agencies. Based on analyses from a scientific workshop sponsored by the National Kidney Foundation and US Food and Drug Administration, which critically examined available data to determine whether alternative GFR‐based endpoints could be used in clinical trials,^4^ a pre‐specified exploratory analysis of VERTIS CV data was conducted that reported a significant risk reduction in a composite kidney outcome (sustained ≥40% decline in eGFR/chronic kidney dialysis/transplantation/renal death [HR 0.66, 95% CI 0.50–0.88]).^5^

We performed a meta‐analysis of kidney composite outcomes from four SGLT2 inhibitor CVOT programs, which included, as a surrogate of progression to kidney failure, a sustained ≥40% decline in eGFR along with kidney replacement therapy and kidney death. The analysis was conducted on the total patient population. The CVOTs assessed were VERTIS CV (NCT01986881),^5^ CANVAS Program (NCT01032629 and NCT01989754),^6^ DECLARE‐TIMI 58 (NCT01730534),^7^ and EMPA‐REG OUTCOME (NCT01131676).^8^ HRs and 95% CIs were pooled with the meta‐analysis performed across trials. A fixed‐effect meta‐analysis approach was used, with heterogeneity assessed using the Cochran Q test statistic and Higgins and Thompson *I* ^2^.^9^, ^10^ Heterogeneity was considered to be low, moderate, or high if *I* ^2^ was less than 25%, 25% to 75%, or greater than 75%, respectively. The HR and its 100 × (1–α) %CI were extracted from the publications of each individual study and converted to log(HR) and its SE before the meta‐analysis. The meta‐analysis was directly implemented on the natural log HR scale, with results exponentiated and reported on the original HR scale. The R package, metafor, version 3.6.2 (R Foundation), was used for all analyses and for forest plot generation. Two‐sided p values <.05 were considered significant.

## 3 RESULTS

**FIGURE 1:**
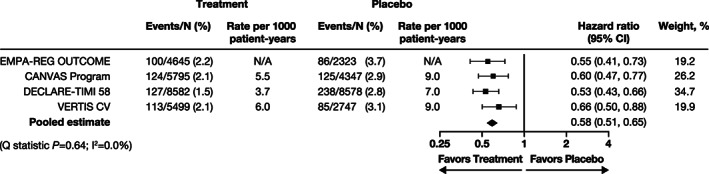
Effects of sodium–glucose cotransporter 2 inhibitors on the kidney composite outcome of sustained* ≥40% decline in eGFR from baseline, kidney replacement therapy, or kidney death. *In the EMPA‐REG OUTCOME and DECLARE‐TIMI 58 trials, 'sustained' was defined as a reduction that was present on at least two consecutive occasions ≥4 weeks apart. In the CANVAS Program and VERTIS CV trial, 'sustained' was defined as a reduction that was present on at least two consecutive occasions >30 days apart. CI, confidence interval; eGFR, estimated glomerular filtration rate. Weights are initially calculated as 1/variance of the hazard ratio and then standardized so that they sum up to 1. Studies with higher precision (i.e. smaller confidence intervals) will be given more weights

Data from the four cardiovascular outcome study programs with 42 516 patients were included. The median follow‐up period for each trial ranged from 2.4 to 4.2 years. A summary of the key baseline characteristics of these trials can be found in Table 1. Overall, there were 998 patients with composite kidney events.^5^, ^6^, ^7^, ^8^ Of these, 947 patients had a sustained 40% decline in eGFR (Table 1). The results of meta‐analysis of effects of SGLT2 inhibitors on the hazard for the kidney composite are presented in Figure 1. Overall, SGLT2 inhibition was associated with a significant reduction in the kidney composite endpoint (HR 0.58, 95% CI 0.51–0.65) and with a highly consistent effect across the trials, with low heterogeneity (Q statistic p = 0.64; *I* ^2^ = 0.0%).

**TABLE 1:** Key baseline characteristics, follow‐up, and number of patients with kidney events for the four SGLT2 inhibitor cardiovascular outcome trials identified for the meta analysis

	EMPA‐REG OUTCOME^8^, ^13^, ^14^	CANVAS Program^6^, ^15^	DECLARE‐TIMI 58^7^, ^16^	VERTIS CV^2^, ^5^
Number of patients	7020	10 142	17 160	8246
Mean age (years)	63	63	64	64
Female (%)	29	36	37	30
Caucasian (%)	72	78	80	88
ASCVD (%)	100	66	41	100
Mean HbA1c (%)	8.1	8.2	8.3	8.2
Mean BMI (kg/m^2^)	31	32	32	32
Mean SBP (mmHg)	135	137	135	133
Mean eGFR (ml/min/1.73 m)	74	77	85	76
Mean duration of follow‐up (years)	NA	3.6	NA	3.5
Median duration of follow‐up (years)	3.1	2.4	4.2	3.0
Number of patients with composite kidney events (n)	186	249	365	198
Sustained 40% decline in eGFR (n)	175	239	341	192
Kidney replacement therapy (n)	25	18	25	12
Kidney death (n)	NA	3	16	2

Abbreviations: ASCVD, atherosclerotic cardiovascular disease; BMI, body mass index; eGFR, estimated glomerular filtration rate; HbA1c, glycated hemoglobin; SBP, systolic blood pressure.

## 4 DISCUSSION

This meta‐analysis highlights the value of using similarly defined endpoints across trials. In previous meta‐analyses,^1^ individual trials used different definitions of significant eGFR loss as a surrogate for adverse kidney outcomes. In VERTIS CV, it was doubling of serum creatinine^2^; in EMPA‐REG OUTCOME,^11^ it was doubling of serum creatinine accompanied with an eGFR of ≤45 ml/min per 1.73 m^2^; in the CANVAS Program,^6^ it was a sustained ≥40% decline in eGFR; and in DECLARE‐TIMI 58,^7^ it was a sustained ≥40% decline in eGFR accompanied by an eGFR of <60 ml/min per 1.73 m^2^. In the current meta‐analysis, the surrogate endpoint of adverse kidney outcomes was sustained ≥40% decline in eGFR (in one trial, the decline in eGFR had to be accompanied with an eGFR of <60 ml/min per 1.73 m^2^).^7^ When using similar definitions, the results of the current meta‐analysis demonstrate a very low measure of heterogeneity, with an *I* ^2^ of 0.0% compared with 49.7% in the prior meta‐analysis using disparate endpoints.^1^

Based on recent literature, an international consensus on definitions of endpoints for clinical trials of kidney failure has emerged, coming to the conclusion that the preferred threshold for kidney function loss is a sustained ≥40% decline in eGFR from baseline.^3^ This threshold of kidney function loss is clinically meaningful, predictive of future need for kidney replacement therapy, and can be captured within the relatively brief timeframe of outcome trials.

SGLT2 inhibitors decrease the risk for kidney composite outcomes in CVOTs, with similar benefits reported in dedicated kidney outcome trials such as CREDENCE and DAPA‐CKD. Based on the results from the SGLT2 inhibitor CVOTs and kidney outcome studies, the Kidney Disease Improving Global Outcomes organization modified their 2020 guidelines for the management of chronic kidney disease in patients with diabetes, leading to the following recommendation “…glycemic management for patients with type 2 diabetes and chronic kidney disease should include lifestyle therapy, first‐line treatment with metformin and an SGLT2 inhibitor, and additional drug therapy as needed for glycemic control”.^12^

## 5 CONCLUSION

This meta‐analysis, using a consistent endpoint, supports the robustness of the finding of reduced kidney disease progression across the class of SGLT2 inhibitors in patients with type 2 diabetes either with established ASCVD or at high cardiovascular risk.

## CONFLICT OF INTEREST

David Z.I. Cherney has received consulting fees or speaking honoraria, or both, from Bristol Myers Squibb, Novo Nordisk, Mitsubishis‐Tanabe, MAZE, Janssen, Bayer, Boehringer Ingelheim‐Eli Lilly, AstraZeneca, Merck & Co., Inc., Prometic, and Sanofi; and has received operating funds from Janssen, Boehringer Ingelheim‐Eli Lilly, Sanofi, AstraZeneca, and Merck & Co., Inc. Samuel Dagogo‐Jack has led clinical trials for AstraZeneca, Novo Nordisk, Inc., and Boehringer Ingelheim; has received fees from AstraZeneca, Boehringer Ingelheim, Janssen, Merck & Co., Inc., and Sanofi; and holds equity interests in Jana Care, Inc. and Aerami Therapeutics. Darren K. McGuire has had leadership roles in clinical trials for AstraZeneca, Boehringer Ingelheim, Eisai, Esperion, GlaxoSmithKline, Janssen, Lexicon, Merck & Co., Inc., Novo Nordisk, CSL Behring, and Sanofi USA; and has received consultancy fees from AstraZeneca, Boehringer Ingelheim, Lilly USA, Merck & Co., Inc., Pfizer, Novo Nordisk, Metavant, Afimmune, and Sanofi. Francesco Cosentino has received fees from Abbott, AstraZeneca, Bayer, Bristol Myers Squibb, Merck Sharp & Dohme, Boehringer Ingelheim, Novo Nordisk, and Pfizer; as well as research grants from Swedish Research Council, Swedish Heart & Lung Foundation, and the King Gustav V and Queen Victoria Foundation. Richard Pratley has received the following (directed to his institution): speaker fees from Novo Nordisk; consulting fees from Merck & Co., Inc., Novo Nordisk, Pfizer, Sanofi, Scohia Pharma Inc., and Sun Pharmaceutical Industries; and grants from Lexicon Pharmaceuticals, Hanmi Pharmaceutical Co., Novo Nordisk, Poxel SA, and Sanofi. Weichung J. Shih has received fees for an ertugliflozin advisory board of Merck & Co., Inc., Kenilworth, New Jersey, USA. Robert Frederich and Shuai Wang are employees and shareholders of Pfizer Inc. Mario Maldonado is an employee of MSD UK. He may own stock and/or stock options in Merck & Co., Inc., Kenilworth, New Jersey, USA. Jie Liu is an employee of Merck Sharp & Dohme Corp., a subsidiary of Merck & Co., Inc., Kenilworth, New Jersey, USA, and may own stock and/or stock options in Merck & Co., Inc., Kenilworth, New Jersey, USA. Christopher P. Cannon reports grants and personal fees from Pfizer Inc., Amgen, Boehringer Ingelheim, Bristol Myers Squibb, and Janssen; grants and personal fees from Merck & Co., Inc., during the conduct of the trial; grants from Daiichi Sankyo and Novo Nordisk; and personal fees from Aegerion, Alnylam, Amarin, Applied Therapeutics, Ascendia, Corvidia, HLS Therapeutics, Innovent, Kowa, Sanofi, Eli Lilly, and Rhoshan, outside the submitted work.

## AUTHOR CONTRIBUTIONS

David Z.I. Cherney, Samuel Dagogo‐Jack, Darren K. McGuire, Richard Pratley, Francesco Cosentino, Mario Maldonado, Jie Liu, and Christopher P. Cannon substantially contributed to the conception, design, or planning of the study. Darren K. McGuire, Robert Frederich, Shuai Wang, and Christopher P. Cannon substantially contributed to the acquisition of the data. Samuel Dagogo‐Jack, Darren K. McGuire, Mario Maldonado, Jie Liu, Shuai Wang, and Christopher P. Cannon substantially contributed to the analysis of the data. David Z.I. Cherney, Samuel Dagogo‐Jack, and Mario Maldonado substantially contributed to the drafting of the manuscript. All authors substantially contributed to the interpretation of the results and critically reviewed and revised the manuscript for important intellectual content.

## Data Availability

Merck Sharp & Dohme Corp., a subsidiary of Merck & Co., Inc., Kenilworth, NJ, USA's data sharing policy, including restrictions, is available at http://engagezone.msd.com/ds_documentation.php. Requests for access to the clinical study data can be submitted through the EngageZone site or via email to dataaccess@merck.com.
